# Nanopriming-mediated memory imprints reduce salt toxicity in wheat seedlings by modulating physiobiochemical attributes

**DOI:** 10.1186/s12870-022-03912-2

**Published:** 2022-11-22

**Authors:** Tahir Farooq, Muhammad Naveed Akram, Amjad Hameed, Toheed Ahmed, Arruje Hameed

**Affiliations:** 1 Department of Applied Chemistry, Government College University, Faisalabad, Pakistan; 2 Department of Biochemistry, Government College University, Faisalabad, Pakistan; 3 Plant Breeding & Genetics Division, Nuclear Institute for Agriculture and Biology (NIAB), Jhang Road, Faisalabad, Pakistan; 4 Department of Chemistry, Riphah International University, Faisalabad, 38000 Pakistan

**Keywords:** Seed priming, Stress acclimation, Salinity, Stress tolerance, Wheat

## Abstract

**Background:**

Around the globe, salinity is one of the serious environmental stresses which negatively affect rapid seed germination, uniform seedling establishment and plant developments restricting sustainable agricultural productivity. In recent years, the concepts of sustainable agriculture and cleaner production strategy have emphasized the introduction of greener agrochemicals using biocompatible and natural sources to maximize crop yield with minimum ecotoxicological effects. Over the last decade, the emergence of nanotechnology as a forefront of interdisciplinary science has introduced nanomaterials as fast-acting plant growth-promoting agents.

**Results:**

Herein, we report the preparation of nanocomposite using chitosan and green tea (CS-GTE NC) as an ecofriendly nanopriming agent to elicit salt stress tolerance through priming imprints. The CS-GTE NC-primed (0.02, 0.04 and 0.06%), hydroprimed and non-primed (control) wheat seeds were germinated under normal and salt stress (150 mM NaCl) conditions. The seedlings developed from aforesaid seeds were used for physiological, biochemical and germination studies. The priming treatments increased protein contents (10–12%), photosynthetic pigments (Chl *a* (4–6%), Chl *b* (34–36%), Total Chl (7–14%) and upregulated the machinery of antioxidants (CAT (26–42%), POD (22–43%)) in wheat seedlings under stress conditions. It also reduced MDA contents (65–75%) and regulated ROS production resulting in improved membrane stability. The priming-mediated alterations in biochemical attributes resulted in improved final germination (20–22%), vigor (4–11%) and germination index (6–13%) under both conditions. It reduced mean germination time significantly, establishing the stress-insulating role of the nanocomposite. The improvement of germination parameters validated the stimulation of priming memory in composite-treated seeds.

**Conclusion:**

Pre-treatment of seeds with nanocomposite enables them to counter salinity at the seedling development stage by means of priming memory warranting sustainable plant growth and high crop productivity.

## Introduction

Sustainable agricultural productivity is under severe threat due to environmental stresses and ecotoxicological conditions caused by global climate change. Collectively, they pose serious challenges to the productivity of high-demand and economically important cereal crops like wheat and highlight the risk to food security [1–3]. Among them, salinity is one of the major issues that reduce plant growth and crop productivity in arid and semiarid regions around the world [4]. Salinity causes oxidative stress, ion toxicity and reduces the water uptake resulting in delayed germination and limited growth. The imbalance in water uptake may result in the closure of stomata and reduced photosynthetic activity. At the same time, salt-mediated ion toxicity disrupts cellular integrity, disturbs the homeostasis of essential ions and influences metabolic activities [5]. Salt stress delays seed germination by damaging abscisic acid/gibberellin balance, respiration processes and energy production. Thus, salt-induced disruptions in physiological and biochemical attributes put negative impacts on the seed germination process, seedling establishment and plant growth, finally reducing crop productivity [6–8].

Wheat is a high-demand food item and an economically important crop, especially in Asian countries. Its yield, productivity and grain quality are often hampered by abiotic stresses. Its seed germination, seedling development and growing plants do experience the same aforementioned negatives on physiological processes and biochemical pathways at subcellular under salt stress [9, 10]. Plants have developed various mechanistic approaches as counteracting strategies to mitigate the damaging effects of salinity. As a protective measure, they try to execute a controlled uptake and selective transportation of desired ions, readjustment of photosynthetic activities and regulation of osmotic potential at the cellular and whole-plant levels. They also reprogram the synthesis of growth-stimulating phytohormones, availability of compatible solutes and management of oxidative stress [11, 12]. Saline conditions negatively influence rapid seed germination and uniform seedling development therefore, seed priming has emerged as a powerful seed-pretreatment approach enabling it to acclimatize stress conditions. It capacitates the seeds to resist environmental stresses by increasing metabolite contents, upregulating antioxidant potential and triggering metabolic changes for final high crop yield [13]*.* Pre-treatments with growth-stimulating agents and subsequent exposure to harsh factors invoke stress memory in primed seeds. The memory imprints help the primed seeds to adopt stressful environment at germination, seedling establishment and plant growth levels with minimum negative impacts [14]. A number of chemical, biological and inorganic compounds have been employed as priming agents showing variable growth-promoting and stress insulating potential [15]. Over the last two decades, the concepts of sustainable agriculture and cleaner production strategy have emphasized the introduction of greener agrochemicals to maximize crop yield with minimum ecotoxicological effects. Over the last decade, the emergence of nanotechnology as a forefront of interdisciplinary science has introduced nanomaterials especially the metallic nanoparticles (NPs) as fast-acting growth-promoting agents and stress emulators [16, 17]. Priming with ZnONPs reduced salt toxicity in wheat plants by regulating antioxidants, photosynthetic electron transport chain and trapped energy flux. The priming treatments also upregulated the enzymes involved in the sucrose biosynthesis in leaves to enhance photosynthetic carbon assimilation as a protective approach for the mitigation of salinity related hazardous effects [18]. According to another study, ZnONPs priming mitigated the salt-induced toxicity by protecting leaf ultrastructure, photosynthetic attributes and electrophoretic profiles of polypeptides in salt-affected wheat plants [19]. Rapeseeds primed with CeONPs coated with polyacrylic acid exhibited increased biosynthesis of salicylic acid and ROS scavenging potential as a salt-stress counteracting strategy [20]. Barely seedlings originated from silicon-primed seeds exhibited upregulated antioxidant defence for early ROS detoxification as a protective strategy against salt stress [21]. The pre-sowing treatments of seeds with AgNPs induced salt tolerance in wheat plants by regulating metabolic pathways and improving enzymatic and non-enzymatic antioxidants [22]. Priming treatments with nano-iron (III) oxide induced salt tolerance in sorghum by increasing relative water content, photosynthetic rate and pigment contents. The nanopriming also enhanced the efficiency of photosystem II as a protective measure to mitigate the hazardous impacts of salinity [23]. However, metal-based nanoformulations have shown few concerns about their applications as fertilizers, fungicides and pesticides etc. [24]. Therefore, it has been emphasized recently to develop nontoxic nanoagrochemicals using sustainable resources to transform traditional agro-practices [25–27].

Considering priming as a facile approach and the high efficiency of nanomaterials, we hypothesized that a biodegradable and nontoxic biopolymer and natural phytochemicals could be exploited as sustainable resources to prepare nanocomposite as an environment-friendly priming agent. Chitosan (CS) is a biodegradable biopolymer, a heterogeneous and cationic polysaccharide that regulates immune signaling, carbon and nitrogen metabolism in plants thus stimulate plant growth and elicit stress resistance [28–30]. Green tea is enriched with polyphenols predominantly catechins which possess a broad spectrum of biological activities including high antioxidant potential [31]. Accordingly, we employed CS and aqueous green tea extract (GTE) as biocompatible and sustainable sources for the preparation of CS-GTE NCas an eco-friendly nanopriming agent. Herein, we report the preparation of CS-GTE NC and its subsequent application for nanopriming of wheat seeds, highlighting its growth-stimulatory and stress emulating role at seed germination and seedling establishment stage under salinity conditions. The study aimed to introduce eco-friendly nanosystem for the induction of salinity tolerance to manifest sustainable crop production.

## Material and methods

### Preparation and characterization of nanocomposite

A 0.2% acetic acid solution was used to prepare 0.1% chitosan solution after stirring for 12 h. In the subsequent step, green tea extract (12 mg) was added to receive a homogenized mixture as solution 1 (S1). On parallel, 0.1% tripolyphosphate solution was prepared as solution 2 (S2). Then, 20 ml of S1 and 5 ml of S2 were mixed and stirred for 2 h before subjecting to centrifugation. Finally, the received nanocomposite was dried and used for characterization and priming studies [32].

**Fig. 1:**
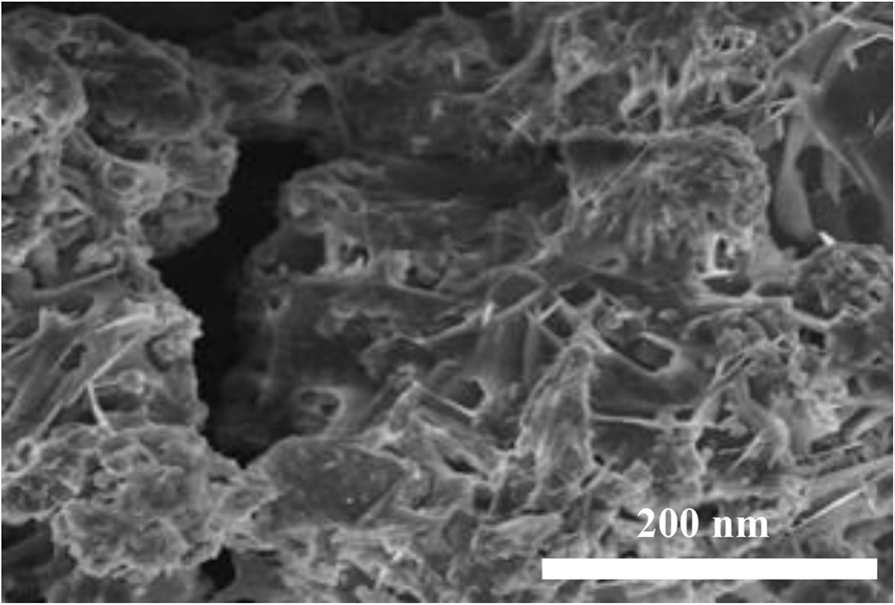
SEM micrograph of the composite of chitosan and green tea extract

**Fig. 2:**
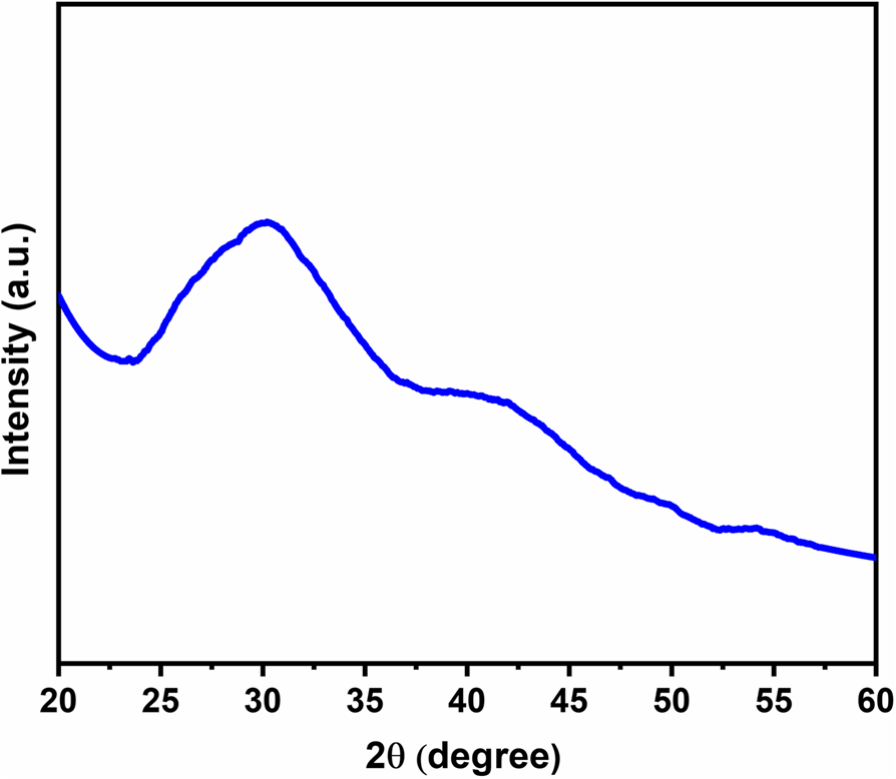
XRD spectra of the composite of chitosan and green tea extract

**Fig. 3:**
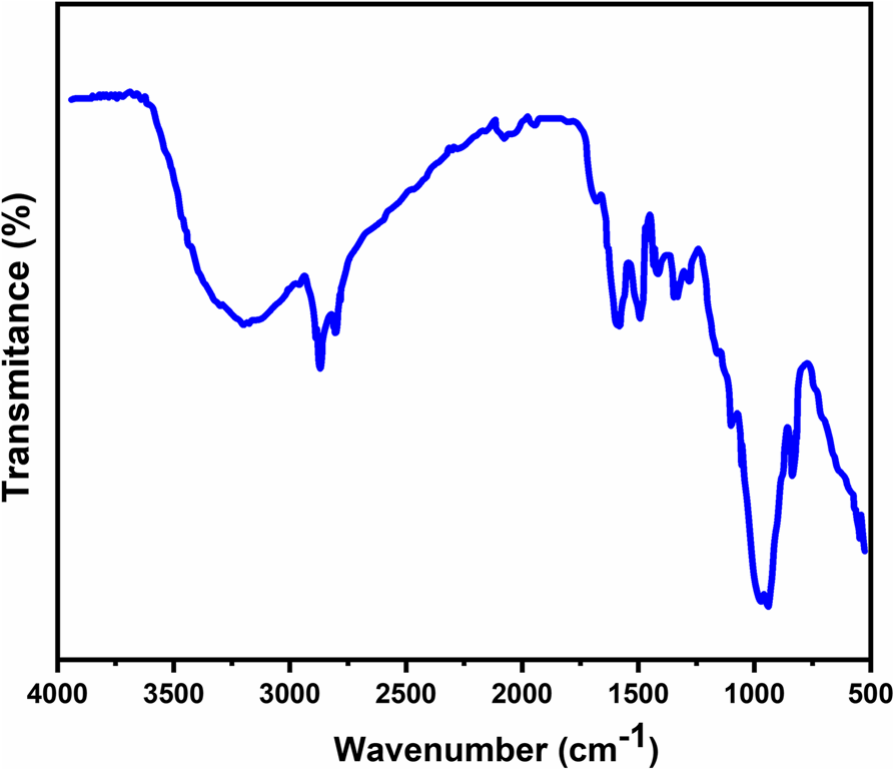
FTIR spectra of the composite of chitosan and green tea extract

The Fig. 1 shows the uniform coating of chitosan and green tea nanocomposites with size ranging 200 nm. The chitosan obtained showed crystalline nature because in Fig. 2 it exhibited a peak at 2θ = 20.3° which is associated to the reflection plane of (200). Further, a predominant amorphous nature has been attributed to a broad region ranging from 20.3 to 80°. The hydrogen bonding capacity of chitosan originates free-energy balance which supports its semi-crystalline nature [33]. The GTE did not show any crystalline peaks. The prepared nanocomposite showed characteristics of an amorphous structure due to the absence of diffraction peaks [34]. The nature of bonding and functional groups was studied using FTIR spectroscopy (Fig. 3). The chitosan, green tea extract and the prepared nanocomposite show moderately different FT-IR spectrum, indicating the composite formation between CS and GTE. A decrease of free –NH_2_ groups exhibited by lessening of peak at 3425 cm^−1^ represents their interactions with polyphenols and confirm the composite formation [35].

### Seed priming and germination studies

The spring wheat (*Triticum aestivum* L. cv. AARI-2011) seeds were received from Ayub Agriculture Research Institute, Faisalabad, Pakistan. The seeds were primed with 0.02, 0.04 and 0.06% of prepared nanocomposite for 8 h. After that, treated seeds were given water-wash and re-dried at 25 ± 2 °C under shade. At the same time, a few seeds were subjected to hydropriming for comparative studies. A completely randomized design was applied and the experiment was run in three replicates to study priming effects on seed germination and seedling development under normal and salt stress conditions. Germination potential of the primed and non-primed seeds was estimated in accordance with the International Rules for Seed Testing by Association of Official Seeds Analyst (AOSA) (Anonymous. 1990). To test seed germination and seedling vigor, three replicates of 24 seeds were germinated in 12 cm diameter petri dishes at 26 °C under normal and salt stress condition. The salt stress was imposed using 150 mM NaCl [22, 23]. A seed was scored as germinated when coleoptile and radicle lengths reached 2–3 mm. Counts of germinating seeds were made twice a day at different time intervals starting on the first day of imbibition, and terminated when maximum germination was achieved.

Seedling samples were collected from non-primed, CS-GTE NC-primed and hydro-primed seeds after 14 days of germination both under normal and stress conditions. Afterward, seedling samples were stored at -80 °C till further analyses.

### Biochemical analyses

Specific extraction buffers were used to grind seedling samples (0.5 g) and subjected to centrifugation at 12,000 × g for 15 min. Then, the supernatant was used for biochemical analyses by following known spectrophotometric procedures (Hitachi U-2800 spectrophotometer) [36].

#### Enzymatic and non-enzymatic antioxidants

One unit of superoxide dismutase (SOD) activity means the amount of enzyme which caused 50% inhibition of photochemical reduction of nitroblue tetrazolium (NBT) dye. Accordingly, the SOD activity was analyzed by measuring its ability to inhibit the photochemical reduction of NBT [37]. Further, an established method was followed for the measurement of catalase (CAT) and peroxidase (POD) activity [38]. One unit of POD and CAT activity was recorded from an absorbance variation of 0.01 units/min. The POD activity was measured by noting the increase in absorbance of the reaction solution at 470 nm. Whereas a decrease in absorbance of the reaction solution at 240 nm provided CAT activity. Further, the enzymatic activities were expressed on a seed weight basis.

The non-enzymatic antioxidants, the total phenolic contents (TPC), were measured following microcolorimetric method using Folin–Ciocalteau reagent [39]. During the analysis, ice-cold methanol was used to homogenize 0.5 g of the sample followed by incubation in dark for 48 h before centrifugation at 12,000 × g for 15 min. Successively, 100 µl of 10% F–C reagent was mixed with 100 µl supernatant before adding 800 µl of 700 mM sod. carbonate sol. Then sample was subjected to incubation for 1 h at room temp. The absorbance of the blank sample was recorded at 765 nm. Different concentrations of gallic acid were used for the preparation of standard curve and the linear regression equation provided TPC (gallic acid equivalents).

#### Malondialdehyde contents

The method based on thiobarbituric acid (TBA) reaction was followed to measure malondialdehyde (MDA) contents for the assessment of the level of lipid peroxidation. For this analysis, 0.1% trichloroacetic acid (TCA) was used to homogenize 0.2 g of the sample and subjected to centrifugation 12,000 × g for 10 min. Then, 0.05% TBA in 20% TCA was added to supernatant before heating to 90 °C for 25 min. Subsequently, the absorbance was recorded at 532 nm and MDA was calculated using 155 mM^−1^ cm^–1^ as extinction coefficient [40].

#### Pigments contents

The carotenoids and photosynthetic pigments (chlorophyll a, b and total chlorophyll) were measured by following known spectroscopic methods [41, 42]. The pigments were extracted in acetone and subjected to centrifugation. The absorbance of the supernatant was taken at 480, 645 and 663 nm. The pigment contents were calculated as followings,

$$\mathrm{Chl}\;\mathrm a\;\left(\mathrm{mg}/\mathrm{gf}.\mathrm{wt}.\right)=\lbrack12.7\left(\mathrm{OD}663\right)-2.69\left(\mathrm{OD}645\right)\times\mathrm V/1000\times\mathrm W\rbrack$$

$$\mathrm{Chl\;b}\left(\mathrm{mg}/\mathrm{g f}.\mathrm{wt}.\right)=[22.9\left(\mathrm{OD }646\right)-4.68\left(\mathrm{OD }663\right) \times \mathrm{V}/1000\times \mathrm{W}]$$

$$\mathrm{Caratenoids }\left(\mathrm{mg}/\mathrm{g f}.\mathrm{wt}.\right)=[\mathrm{Acar}/\mathrm{EM}]\times 1000\mathrm{ Acar }=\mathrm{OD }480+0.114(\mathrm{OD }663)-0.638(\mathrm{OD}645)$$

where OD represents optical density, V is the volume of the sample, W is the weight of fresh tissue taken for extraction and EM is 250.

### Germination parameters

The aforementioned germinating seeds were used to calculate final germination, mean germination, germination energy, germination index and vigor index.

#### Mean germination time

Mean germination time (MGT) was calculated according to the following Eq. [43].

$$\mathrm{MGT }= \sum \mathrm{ Dn }/ \sum \mathrm{ n}$$

where n is the number of seeds, which were germinated on day D and D is the number of days counted from the beginning of germination.

#### Final germination percentage

Final germination percentage was measured according to following formula.

$$\mathrm{FGP}=\mathrm{No}\;\mathrm{of}\;\mathrm{seeds}\;\mathrm{germinated}\;\mathrm{on}\;\mathrm{final}\;\mathrm{day}/\mathrm{Total}\;\mathrm{no}\;\mathrm{of}\;\mathrm{seeds}\;\mathrm{sown}\times100$$

#### Germination index

Germination index (GI) was calculated as described by the Association of Official Seed Analysts (AOSA) [44] using the following formula.

$$\mathrm{GI}=\mathrm{ number of germinated seeds}/\mathrm{Days of first count }+ -------------+\mathrm{ number of germinated seeds}/\mathrm{days of final count}$$

#### Energy of germination

Energy of germination was recorded 4th day after planting. It is the percentage of germinated seeds 4 days after planting relative to the total number of seeds tested [45].

### Statistical analyses

The analyses of variance and Tukey (HSD) Test at *p* < 0.05 were used to measure the significance of data using XL-STAT software (version 2012.1.02) by Addinsoft (www.xlstat.com) and the mean ± SD values are given in tables/figs.

## Results

The non-primed, hydroprimed and CS-GTE NC-primed seeds were germinated under normal and stress conditions in separate petri dishes. Subsequently, the seedlings originated from the aforementioned seeds were employed for physiological, biochemical and germination studies and compared with controls (non-primed).

### Effects of nanopriming on antioxidants and lipid peroxidation

Under control conditions, a little increase in CAT activity was observed in seedlings originating from primed seeds. A significant increase in CAT (26–42%) activity was recorded under stress however, the increasing effect decreased with increasing priming concentration (Table 1). The priming treatments significantly increased APX activity under normal 43–52% and stress conditions (7–14%) (Table 1). The POD activity increased significantly with a concentration-dependent increasing and decreasing effect under stress (22–43%) and non-stress condition (22–50%) respectively (Table 1). Under both conditions, the priming did not induce any significant change in SOD activity (Table 1).

**Table 1:** Effect of Green tea extract priming treatments on enzymatic antioxidants in wheat seedlings under salt stress

Enzymatic Antioxidants	Treatments									
	**Non- Stress**					**Under Salt Stress (150 mM NaCl)**				
	**Control**	**Hydro-priming**	**0.02%Green Tea Extract Priming**	**0.04%Green Tea Extract Priming**	**0.06%Green Tea Extract Priming**	**Control**	**Hydro-priming**	**0.02%Green Tea Extract Priming**	**0.04%Green Tea Extract Priming**	**0.06%Green Tea Extract Priming**
**Catalase (CAT)** **(Enzyme units/g FW)**	263 ± 2.082 f	263 ± 1.732 f	286 ± 3.786 d	275 ± 2.082 e	274 ± 2.333 e	263.333 ± 2.028 f	284 ± 2.082 e	405.333 ± 2.728 a	388.333 ± 4.631 b	343 ± 2.082 c
**Peroxidase (POD)** **(Enzyme units/g FW)**	2931.733 ± 0.321 d	3064.567 ± 0.186 c	4929 ± 0.811 a	3798 ± 0.917 b	3670.467 ± 0.404 b	1798.900 ± 4.376 g	1465.567 ± 0.612 h	1803.300 ± 4.419 g	2265.833 ± 0.902 f	2797.100 ± 0.839 e
**Superoxide dismutase (SOD)** **(Enzyme units/g FW)**	233.674 ± 2.021 ab	233.749 ± 1.565 ab	236.008 ± 1.266 ab	240.674 ± 5.600 a	238.495 ± 7.223 ab	207.105 ± 6.170 c	206.873 ± 3.207 c	217.565 ± 4.774 abc	217.105 ± 2.155 bc	209.181 ± 7.147 c
**Ascorbate Peroxidase (APX)** **(Enzyme units/g FW)**	285.333 ± 2.728 f	285 ± 2.082 f	483 ± 2.082 d	487 ± 4.041 d	443 ± 2.646 e	523 ± 2.028 d	527 ± 3.606 e	564 ± 2.309 g	563 ± 2.028 a	606 ± 3.480 c

Mean values of three replicates presented. Within a row, means sharing the same letters are non-significantly different (*P* > 0.05 and *P* > 0.01) according to the Tukey’s Test (HSD)

**Fig. 4:**
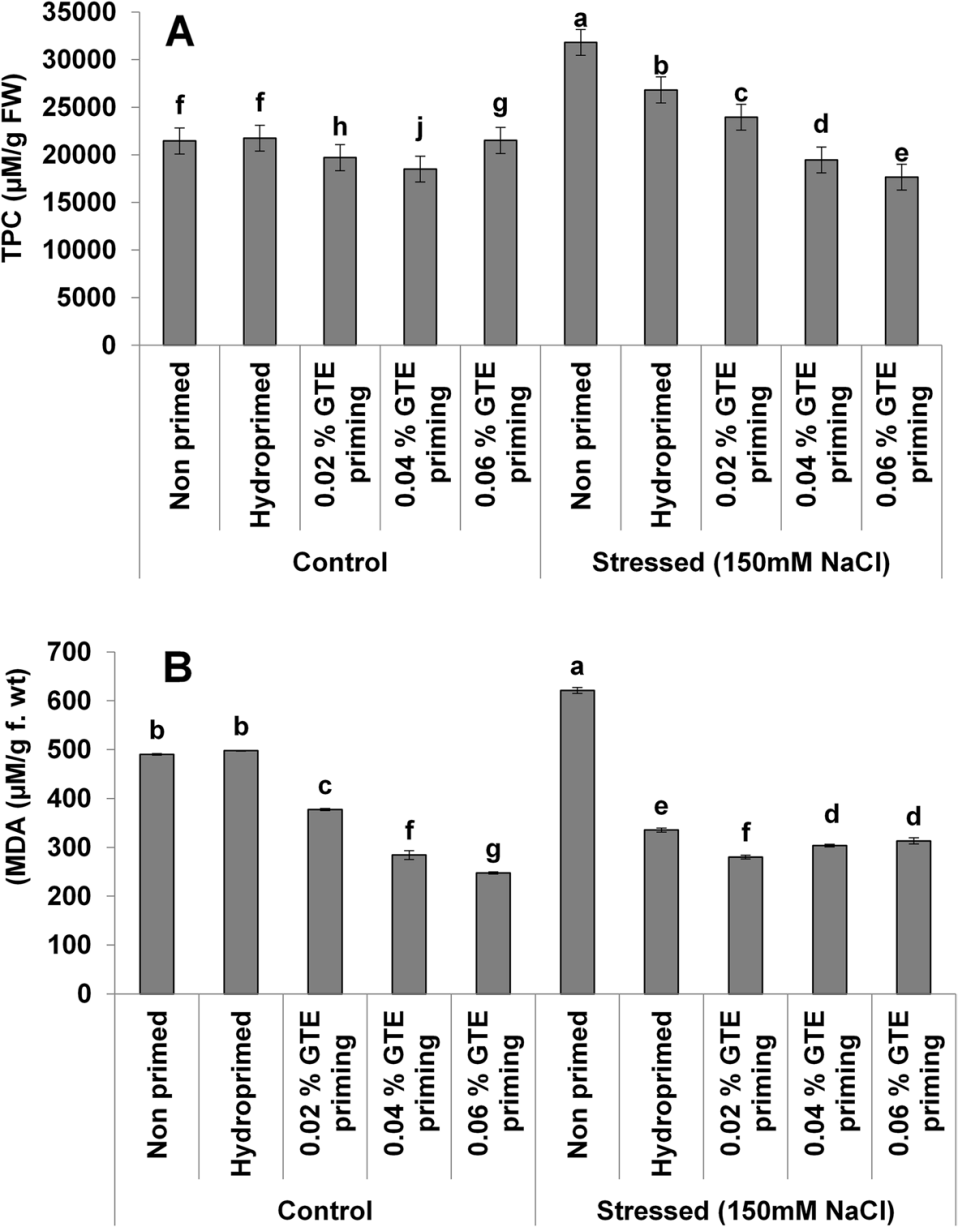
Effect of green tea extract priming on total phenolic (TPC) and MDA contents in wheat seedlings under salt stress

**Fig. 5:**
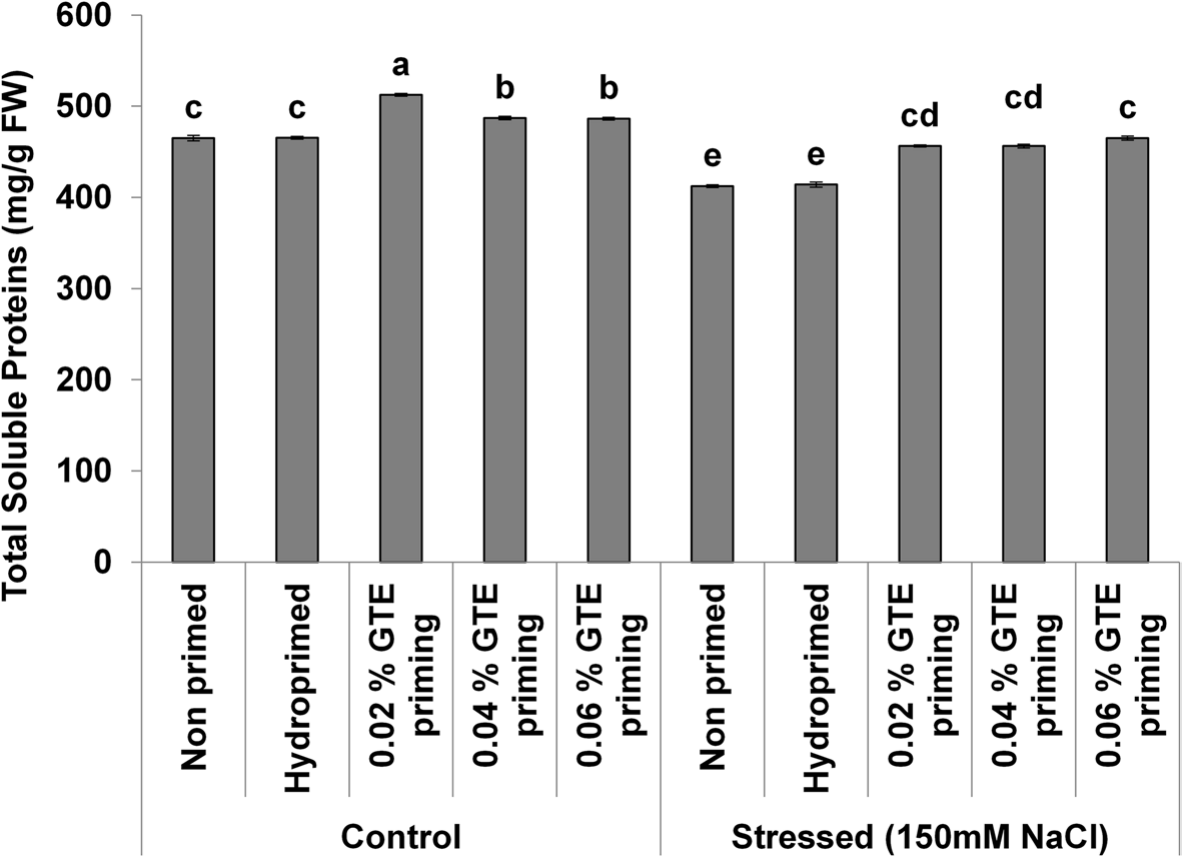
Effect of green tea extract priming on total soluble protein (TSP) contents in wheat seedlings under salt stress

**Fig. 6:**
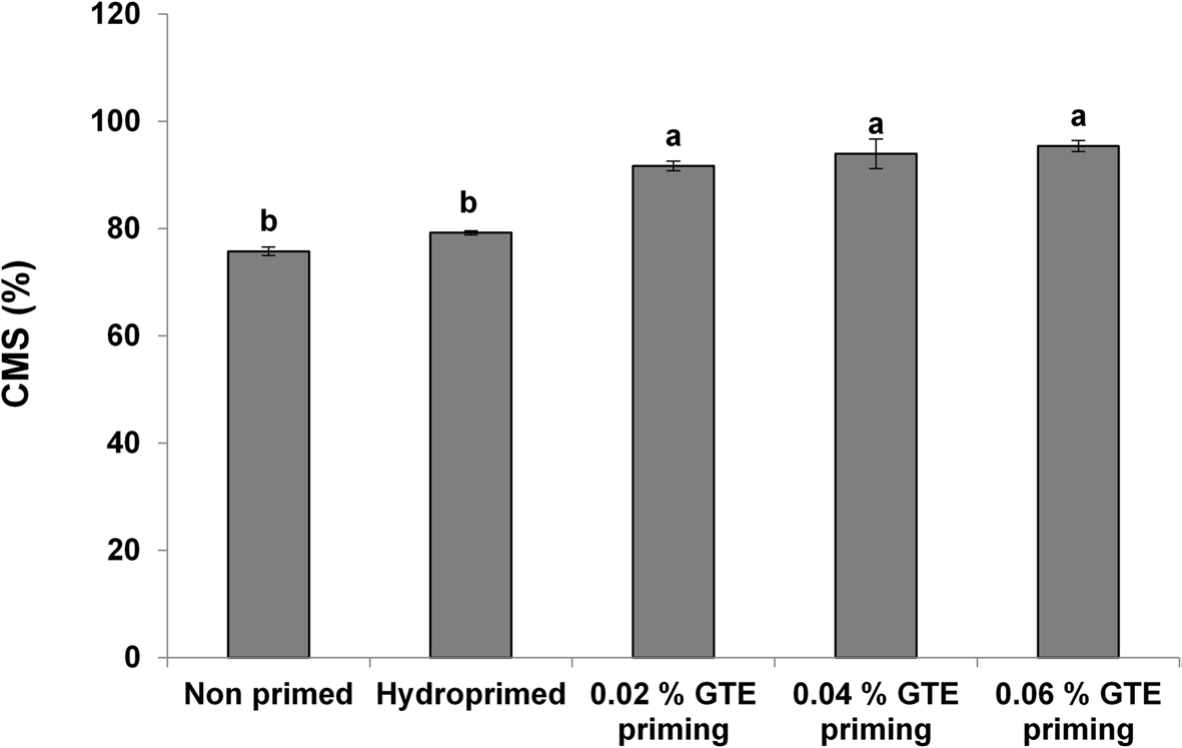
Effect of green tea extract priming on cell membrane stability in wheat seedlings under salt stress

TPC decreased significantly under normal (8–14%) and (28–57%) stress conditions with a clear concentration-dependent decreasing trend under salt stress (Fig. 4A). The MDA contents decreased [46–56] significantly under normal as well as under stress as a result of priming treatments (Fig. 4B). The priming induced a significant increase in TSP in wheat seedlings under normal (4–9%) and stress (10–12%) (Fig. 5). Cell membrane stability significantly increased after all priming treatments (Fig. 6).

### Effects of nanopriming on photosynthetic pigments

A significant increase in chlorophyll *a* (4–6%), *b* (34–36%) and total chlorophyll (7–14%) were observed under stress conditions (Table 2). However, under normal conditions, chl *a*, chl *b* and total chl increased 11–17%, 3–38% and 8–21% respectively. The carotenoid contents increased significantly (15–18%) under normal conditions and (6–9%) under stress (Table 2). The lycopene contents decreased (29%) significantly with 0.04% priming-treatment only under normal while it showed a significant increase (9%) under stress conditions (Table 2). The tannins decreased significantly under non-stress condition whereas a significant increase was recorded as a result of 0.02% and 0.06% priming under stress (Table 2).

**Table 2:** Effect of Green tea extract priming treatments on pigments in wheat seedlings under salt stress

Pigments	Treatments										
	**Non- Stress**					**Under Salt Stress (150 mM NaCl)**					
	**Control**	**Hydro-priming**	**0.02%Green Tea Extract Priming**	**0.04%Green Tea Extract Priming**	**0.06%Green Tea Extract Priming**		**Control**	**Hydro-priming**	**0.02%Green Tea Extract Priming**	**0.04%Green Tea Extract Priming**	**0.06%Green Tea Extract Priming**
**Chlorophyll a** **(µg/g FW)**	133.655 ± 0.050 c	134.878 ± 0.052 c	149.215 ± 0.388 b	151.357 ± 0.717 b	159.748 ± 0.739 a		125.427 ± 0.259 e	125.030 ± 0.865 e	131.659 ± 0.700 cd	133.206 ± 1.713 c	132.511 ± 1.563 cd
**Chlorophyll b** **(µg/g FW)**	64.382 ± 0.154 d	65.692 ± 0.064 cd	95.400 ± 0.150 a	83.543 ± 0.108 b	66.978 ± 0.836 c		40.622 ± 0.404 g	52.392 ± 0.253 f	58.841 ± 0.710 e	57.473 ± 0.437 e	57.342 ± 0.842 e
**Total chlorophyll** **(µg/g FW)**	198.674 ± 0.130 c	198.643 ± 0.134 c	246.660 ± 0.259 a	215.439 ± 0.460 b	217.066 ± 0.597 b		171.122 ± 0.483 f	175.292 ± 0.250 e	198.632 ± 0.611 c	185.084 ± 0.737 d	184.452 ± 1.038 d
**Total carotenoids** **(mg/g FW)**	12.276 ± 0.194 bc	12.364 ± 0.040 b	14.629 ± 0.112 a	14.387 ± 0.056 a	14.714 ± 0.296 a		11.080 ± 0.138 c	11.812 ± 0.139 bc	11.897 ± 0.290 bc	11.879 ± 0.237 bc	12.222 ± 0.501 bc
**Lycopene contents** **(mg/g FW)**	7.341 ± 0.055 ab	6.314 ± 0.073 bcd	6.553 ± 0.139 bcd	5.434 ± 0.077 e	6.624 ± 0.268 abc		6.077 ± 0.151 cd	6.127 ± 0.278 bcd	6.930 ± 0.290 abc	6.234 ± 0.263 bcd	6.862 ± 0.400 abc

Mean values of three replicates presented. Within a row, means sharing the same letters are non-significantly different (*P* > 0.05 and *P* > 0.01) according to the Tukey’s Test (HSD)

### Effects of nanopriming on germination indices

The nanopriming caused significant improvements (*p* < 0.05) in final germination (8–10% and 20–22%) under normal and stress conditions (Table 3). The mean germination time reduced significantly under both conditions while the influence was more evident (3–7%) under normal condition (Table 3). The priming treatments were unable to induce any significant change in germination energy under both conditions compared to control (Table 3). Whereas, both germination index (6–22%, 6–13%) and vigor index (4–24%, 7–11%) increased significantly under normal as well as stress conditions respectively (Table 3).

**Table 3:** Effect of Green tea extract priming treatments on germination parameters in wheat seedlings under salt stress

Germination Parameters	Treatments									
	**Non- Stress**					**Under Salt Stress (150 mM NaCl)**				
	**Control**	**Hydro-priming**	**0.02%Green Tea Extract Priming**	**0.04%Green Tea Extract Priming**	**0.06%Green Tea Extract Priming**	**Control**	**Hydro-priming**	**0.02%Green Tea Extract Priming**	**0.04%Green Tea Extract Priming**	**0.06%Green Tea Extract Priming**
**Final Germination Percentage (%)**	90 ± 0.082 b	95 ± 0.732 b	98 ± 0.786 a	100 ± 0.082 a	100 ± 0.333 a	80 ± 0.028 c	90 ± 0.082 b	98 ± 2.728 a	97 ± 0.631 a	100 ± 0.082 a
**Mean Germination Time (hours)**	56.425 ± 0.040 a	56.379 ± 0.039 a	52.341 ± 0.062 f	53.139 ± 0.045 e	54.754 ± 0.006 c	55.589 ± 0.001 b	53.848 ± 0.009 d	53.124 ± 0.030 e	53.940 ± 0.023 d	53.944 ± 0.005d
**Germination Energy**	10 ± 0.333 b	10 ± 0.333 a	10 ± 0.333 b	11 ± 0.577 b	10 ± 0.333 b	10 ± 0.333 b	10 ± 0.027 b	10 ± 0.074 b	10 ± 0.015 b	10 ± 0.012 b
**Germination index**	13.859 ± 0.015 f	14.109 ± 0.059 ef	17.305 ± 0.035 a	16.338 ± 0.061 b	14.759 ± 0.102 d	14.334 ± 0.001 e	15.667 ± 0.166 c	16.337 ± 0.004 b	15.330 ± 0.004 c	15.338 ± 0.009 c
**Vigor index**	1.114 ± 0.012 g	1.114 ± 0.009 g	1.167 ± 0.009 f	1.267 ± 0.009 e	1.430 ± 0.006 c	1.457 ± 0.012 c	1.523 ± 0.009 b	1.567 ± 0.009 b	1.527 ± 0.012 b	1.637 ± 0.009 a

Mean values of three replicates presented. Within a column, means sharing the same letters are non-significantly different (*P* > 0.05 and *P* > 0.01) according to the Tukey’s Test (HSD)

## Discussions

Usually, salt stress causes excessive production of reactive oxygen species (ROS) and their teal-time detoxification is managed by germinating seeds and growing plants with boosting of antioxidant potential. Therefore, up-regulated enzymatic antioxidants such as APX, CAT, SOD and POD are correlated as positive counteracting developments for the acclamation of abiotic stresses like salinity [57–59]. The priming as well as foliar applications of chitosan modulate metabolic activities and antioxidant enzymes in germinating seeds and growing plants enabling them to suppress the overproduction of ROS [60–62]. The green tea polyphenols have well-established antioxidant potential. They induce antioxidant effects by different approaches including their direct role as antioxidants, initiation of pro-oxidant reactions or by increasing antioxidant enzymes [63, 64] In the direct approach, the free radicals are eliminated when they react with polyphenols forming relatively stable phenolic-oxygen radicals. The singlet electron on oxygen is stabilized by conjugation with pi-electrons of the aromatic ring. Thus, polyphenols with more –OH groups show higher antioxidant potential. Green tea polyphenols regulate the expression of enzymatic antioxidants and ensure continuous scavenging of free radicals [65]. Other studies also showed the applications of tea polyphenols upregulated antioxidant enzymes, reduced lipid peroxidation and suppressed oxidative stress [66, 67]. The CS is involved in a number of cascade reactions which control the production of phytohormones and the expression of antioxidant enzymes. Under salinity conditions, the CS applications increased the activities of antioxidant enzymes in milk thistle, egg plants and tomatoes [68, 69]. Likewise in this study, priming with prepared nanocomposite caused a significant increase in POD, APX and CAT activity. The non-enzymatic antioxidant, TPC declined significantly with increasing priming concentration, representing their lesser requirements under the scenario of hyperactive enzymatic antioxidants and due to the availability of tea polyphenols. Thus, the upregulated antioxidants controlled the oxidative stress under normal and stress conditions which is exhibited by lower MDA content, the biomarker of stress injury. Germinating seeds and growing plants accumulate phenolic compounds as a direct salinity-countering strategy. They reduce lipid peroxidation and protect cell membranes by reducing the mobility of free radicals and lowering membrane fluidity. Further, the CS treatments could regulate genes involved in controlling the biosynthesis of phenolics and other secondary metabolites [70].

Rapid seed germination, uniform seedling development and regulated plant growth heavily rely on the availability of proteins. They act as an alternative source of energy and the main reservoir of amino acids at important phases of germination and development. Various proteins as vital enzymes execute several metabolic pathways and signaling processes as stress-insulating approaches. Salinity-mediated reduced water uptake induces negative impacts on enzyme-controlled metabolic steps because they highly depend on water availability [71, 72]. As an adaptive strategy, salinity-responsive proteins are produced to counterbalance damaging impacts on sub-cellular processes and for the induction of stress tolerance through the readjustment of biochemical attributes [73, 74]. The CS applications further boost the production of proteins and other important biomolecules under stress conditions [47, 48]. CS-priming treatments induced an increase in TSP as a salinity-responsive strategy in rice seedlings [48]. The CS applications also mitigated the negative impacts of salinity in tomatoes by regulating the expression of protein patterns. The CS is supposed to regulate the expressions of vital enzymes of glycolysis thus increase protein contents [49]. Accordingly, in our study, the nanopriming significantly increased TSP suggesting it is a progressive response inducing salinity acclimation at the seedling development stage. The increase in TSP has been considered a priming-mediated stress acclamatory response. The produced proteins may have synchronized any dysfunctionalities in metabolic pathways, providing nutrients, or acted as an alternative source of energy.

In general, abiotic stresses like salinity reduce photosynthetic activity by decreasing photosynthetic pigments in plants [50, 51]. The chitosan applications are known to increase the lycopene, carotenoid and chlorophyll contents as a salinity mitigating response [52, 53]. Salt-mediated toxicity induces the accumulation of chlorophyll-degrading enzymes and disrupts the protein complexes vital for photosynthetic activity. However, the CS treatments insulate photosynthetic pigments by protecting the integrity of proteins involved in chlorophyll metabolism and photosynthetic compartments by detoxifying excessive ROS. Further, the tea polyphenols could act as antioxidants directly and may strengthen the protection of photosynthetic apparatus and related protein complexes [54]. In this study, the priming-treatments significantly improved total chlorophyll, chlorophyll *a*, and *b* under both conditions. Also, there was a significant increase in lycopene and carotenoid contents under stress conditions, suggesting a stress acclamatory role of nanopriming. They are considered the main sources for the de novo synthesis of vital accessory photosynthetic pigments and phytohormones [55]. The tannins are also known for their growth-promoting, antioxidant and salt-stress tolerance properties [56]. Therefore, an increase in chlorophyll contents and tannins and no significant change in carotenoids under salinity suggested a stress-insulating property of the prepared nanopriming agent. Under salt stress, the nanopriming caused a significant increase in CMS through a regulated ROS generation, the fact has also been supported by the boosted anti-oxidative enzymes and low MDA contents. Excessive ROS generation could damage cellular organelles, proteins and nucleic acids resulting in abnormal cellular functioning [75, 76]. It is suggested that nanocomposite-based priming has maintained the threshold gradient of ROS, improved osmoregulation and avoided cellular damages by integrating antioxidative machinery for sustainable seedling development. The controlled ROS production favors the smooth execution of signaling processes and metabolic pathways ensuring membrane integrity with induction of stress tolerance [77, 78].

The CS applications are known to interfere positively with a myriad of complex networks including cellular signaling, transcription processes, ionic and water transport, cell redox homeostasis and metabolic activities in germinating seeds. All such CS-mediated physiological and biochemical positive correlations enhance seedling development by minimizing the toxic effects of salt stress [79]. Further, the polyphenols of GTE serve as the first line of defense due to their direct antioxidant role to maintain cellular redox homeostasis for well-regulated metabolic activities for germination and seedling development. In our case, the nanopriming-mediated alterations in biochemical attributes resulted in improved final germination, vigor and germination index under both conditions. At the same time, it reduced mean germination time significantly, establishing the growth-promoting role of nanocomposite. The improvement of germination parameters substantiated the stimulation of priming memory in composite-treated seeds which conferred resistance against salt stress. Therefore, pre-treatment of seeds with nanocomposite enable them to counter salinity at germination and seedling development stages by means of priming-memory imprints.

## Conclusion

The priming treatments with ecofriendly nanocomposite of chitosan and green tea extract increased protein contents (10–12%), photosynthetic pigments (Chl *a* (4–6%), Chl *b* (34–36%), Total Chl (7–14%)and regulated the machinery of antioxidants (CAT (26–42%), POD (22–43%)) in wheat seedlings under salt stress conditions. It also reduced MDA contents (65–75%), regulated ROS production, and improved membrane stability thus facilitated germination process by conferring salinity tolerance. Hence, the nanocomposite-mediated priming imprints elicit salt stress acclimation at seed germination and seedling establishment warranting sustainable plant growth and high crop productivity.

## Data Availability

All data generated or analysed during this study are included in this published article.
